# The Potential of Integrating Provitamin A-Biofortified Maize in Smallholder Farming Systems to Reduce Malnourishment in South Africa

**DOI:** 10.3390/ijerph15040805

**Published:** 2018-04-19

**Authors:** Mthokozisi K. Zuma, Unathi Kolanisi, Albert T. Modi

**Affiliations:** 1 Department of Crop Science, School of Agricultural, Earth and Environmental Sciences, University of KwaZulu-Natal, Private Bag X01, Scottsville 3209, Pietermaritzburg, South Africa; ModiAT@ukzn.ac.za; 2 Faculty of Science and Agriculture, Department of Consumer Sciences, University of Zululand, KwaDlangezwa 3886, South Africa; kolanisiu@unizulu.ac.za

**Keywords:** Provitamin A-biofortified maize, vitamin A deficiency, maize

## Abstract

Biofortification interventions have the potential to combat malnutrition. This review explored the use of provitamin A-biofortified maize (PVABM) as a vitamin A deficiency (VAD) reduction agricultural-based strategy. Maize has been identified as one of the key staple crops for biofortification to reduce hidden hunger in Africa. Most nutrition interventions have not been successful in reducing hunger because rural communities, who mainly rely on agriculture, have been indirectly excluded. The biofortification intervention proposed here aims to be an inclusive strategy, based on smallholder farming systems. Vitamin A is a micronutrient essential for growth, immune function, reproduction and vision, and its deficiency results in VAD. VAD is estimated to affect more than 250 million children in developing countries. In Africa, especially sub-Saharan Africa, maize is a staple food for rural communities, consumed by most household members. Due to carotenoids, PVABM presents an orange color. This color has been reported to lead to negative perceptions about PVABM varieties. The perceived agronomic traits of this maize by smallholder farmers have not been explored. Adoption and utilization of PVABM varieties relies on both acceptable consumer attributes and agronomic traits, including nutritional value. It is therefore important to assess farmers’ perceptions of and willingness to adopt the varieties, and the potential markets for PVABM maize. It is essential to establish on-farm trials and experiments to evaluate the response of PVABM under different climatic conditions, fertilizer levels and soils, and its overall agronomic potential. For the better integration of PVABM with smallholder farming systems, farmer training and workshops about PVABM should be part of any intervention. A holistic approach would enhance farmers’ knowledge about PVABM varieties and that their benefits out-compete other existing maize varieties.

## 1. Introduction

Maize (*Zea mays*, L.), also known as corn, belongs to the grass family Poaceae (*Gramineae*) [1]. Maize is one of the three most important crops worldwide and the major staple crop on the African continent [2]. In the sub-Saharan Africa (SSA) region, maize plays a significant role in reducing poverty and improving the food security status for poor families [3]. In the sub-Saharan region, maize is used for human consumption, while in developed countries it is used for profit making from feed, fuel and other raw materials for industrial products [3,4]. It has been reported that maize alone, without following a diversified diet, encourages food and nutrition insecurity. In most rural communities in South Africa, people consume starchy staple diets made from maize, with limited or no diversification, leading to unbalanced diets [5]. Maize is characterized by anti-nutritional factors, such as phytate, which is a resistant starch that hinders maize nutrient availability for human consumption [6]. This anti-nutritional factor binds essential nutrients, leading to nutrients not being fully accessible or digested, while other factors inhibit certain enzymes needed for normal functioning or the absorption of minerals and vitamins by humans [6]. This could justify the existence of hidden hunger in countries where maize is the staple crop.

Hidden hunger is a global micronutrient disease, of which there are more than 925 million hungry people in the world [7]. In the 21st century, malnutrition mainly manifests itself as hidden hunger, as opposed to under-nutrition, which used to be the case in previous centuries (19th and 20th centuries). Vitamin A deficiency (VAD) has been reported as one of the major micronutrient deficiencies and a challenge beyond the Millennium Developmental Goals (MDGs) [7]. Vitamin A plays a vital role in supporting immune function, vision, ocular health, and reproduction [8]. There are two types of vitamin A: preformed (animal-based) and provitamin A (plant-based) [9]. Provitamin carotenoids in foods such as β-carotene are major sources of vitamin A [10]. Most provitamin A carotenoids are available in green, orange and yellow crop tissues [11,12]. Unfortunately, these food sources are usually costly, thus inaccessible to low income communities [13,14]. Therefore, the enhancement of food crops, especially staple crops, through biofortification can lead to the production of food with sufficient carotenoids to combat VAD.

HarvestPlus organization introduced the biofortification of food plants to improve vitamin A content in staple crops, such as maize, sweet potatoes and wheat [12], with the aim of alleviating hidden hunger in developing countries, such as South Africa. One of the products they produce is provitamin A-biofortified maize (PVABM); a product of breeding maize with a high provitamin A content to combat VAD in Africa. Furthermore, other potential biofortification products are beans (Iron), pearl millet (Iron), cassava (Vitamin A), sweet potatoes (Vitamin A), Rice (Zinc) and Wheat (Zinc).

A number of studies have been conducted looking at farmers’ perceptions, consumer acceptance, breeding and the potential impact of PVABM in combating micronutritional malnutrition. These studies have primarily been conducted in the sub-Saharan region, as per the HarvestPlus programme. Studies conducted on PVABM have shown that there is the potential for this maize to be accepted by rural communities [12]. Along with all the previously noted benefits of PVABM, researchers should not overlook the current smallholder farming systems in rural communities, where farmers utilize the local maize landraces in their marginal agricultural land. Farmers will need to be convinced to introduce PVABM into their farming systems. The aim of this review was to explore the potential for incorporating PVABM into smallholder farming systems. The review firstly focuses on the agronomic characteristics of maize as a staple crop in South Africa. Then, the potential of PVABM for reducing VAD, the nutritive value of PVABM, its drought tolerance, and perceptions surrounding PVABM as a food source for rural households, will be discussed.

## 2. Maize as a Staple Crop in South Africa

Maize (*Zea mays*) is a staple crop in South Africa [13] and a source of carbohydrates for both humans and animals [14]. In South Africa, maize is grown throughout the country under different climatic conditions. The major producing areas of maize are North West, Free State and Mpumalanga, while in Kwazulu-Natal, it is mostly produced for household consumption [14]. Smallholder farmers prefer to produce white maize for their household consumption and sell it as green maize [14]. On the other hand, yellow maize is mostly produced for animal feed and for brewing traditional drinks.

Many smallholder farmers in South Africa still produce local maize landraces [15]. Regardless of the high yielding varieties bred by researchers, these farmers continue to use local varieties in their farming systems. This justifies the importance of choice and farmer preference as a selection criterion for maize variety. Maize landraces have certain characteristics (phenotypical, genotypic and morphological) that allow them to adapt to the different climatic conditions in the country [12]. These characteristics are the motivation smallholder farmers have for keeping their landraces.

Most rural households depend on natural resources for their farming and basic living needs [16] and in rural areas of South Africa, maize is produced under these natural resources. It is only grown during the rainy season (October to April) due to the inability of smallholder farmers to access water for irrigation [17]. Maize in South Africa is usually planted during the growing season, but the specific dates differ with farmer’s preference and area [18]. According to Pillay [19], October and November are optimal planting dates for maize in South Africa. Subsistence farmers wait for the first rains to plant their maize during the growing season because their production is a dryland system. Subsistence farmers hardly take to note maize population and planting densities, however these are key determinants of yield. A high plant population results in plant competition for light and space, which can have a negative impact on plant growth and yield. Over recent years, smallholder farm maize production has been successful and has contributed significantly to household financial income and as a food source. However, nutrient balance remains questionable, given the major reliance on and high consumption of maize by rural households.

## 3. Agronomy Characteristics of Maize Production

### 3.1. Seed Establishment and Maize Growth

Seed establishment consists of three stages: germination, emergence and early establishment [15]. Seed establishment is environmentally sensitive [20]; therefore, knowledge of environmental conditions is important to note before planting because these are the determinants of germination. Seed characteristics are an important determinant of seed establishment and the environments the seed can establish in. Seed establishment is an important determinant of potential planting date. Poor germination and seedling growth can lead to poor maize grain yields [1]. Plant establishment influences the growth of a maize crop. Poor maize establishment can result in low maize grain yields. Therefore, it is critical for PVABM maize to have good seed establishment in order to produce better maize yields. Higher PVABM grain yields could reduce the existence of hidden hunger and VAD in South Africa.

### 3.2. Plant Density, Planting Date and Maize Production

The best maize planting conditions are frost-free environments with warm temperatures and high altitudes areas [19]. Mazvimbakupa et al. [1] found that average maize yield in high altitude areas was higher than the tropics under field conditions. Climatic factors and genetic variations play a huge role, from plant growth to maize yield. Maize thrives in well-drained soils, but it can also be produced in well-aerated loam and sandy loam soils [19]. Maize has high nutrient demands and the crop is sensitive to soil acidity [18]. Maize is also sensitive to nutrient deficiencies in the soil (e.g., nitrogen, phosphorus and potassium). According to Odendo and Odongo [4], farmers from low income communities tend to use maize–legume intercropping to improve nutrient availability in the soil. Legumes are known to fix nitrogen in the soil and, therefore, their intercropping benefits maize. Farmers use different intercropping systems as a strategy to avail nutrients and reduce pest populations; these mixtures may be bean–maize or cowpea–maize [21].

### 3.3. Effects of Drought on Maize Production

Plant growth is influenced by several factors, including soil fertility, variety, environment, plant density and planting date [22]. Therefore, it is important to understand how physiological and morphological interactions occur in a plant in certain environments and to understand how to apply proper management practices for better growth and maximized grain yields. Vegetative growth of maize is sensitive to drought, just like other grass species and this sensitivity can lead to reduced growth [22]. Less grain yield can be expected when maize is produced under water stressed conditions.

South Africa is a water-scarce country [23] and maize is sensitive to drought during growth [14]. This is one of the key limiting factors for rain-fed agriculture, which is usually the type of agriculture practiced by smallholder farmers in low income communities. Drought can have a large influence on plant performance as it can affect germination duration and growth rate, and can have a negative impact on seedling establishment [15]. Poor seed establishment and poor growth results in a decline in maize grain yield. As a possible solution to drought stress, the use of cultivars with improved drought tolerance may be the only affordable option for many small-scale farmers [24]. Cakir [25] showed that water deficiency strongly affects the different growth stages of corn and that the degree of yield reduction depends on the severity of the water stress. The instability of maize yields caused by drought can have impact on food security at a household level, given the important of maize as a staple crop in rural communities. Similar to maize growth, grain yield is also susceptible to drought [15]; therefore, different yields can be expected for different maize varieties, including the drought resistant breeds.

Global climate change could lead to temperature rises and changes in rainfall distribution [25]. This could produce significant yield losses as a result of drought, especially for smallholder farmers in low income communities who do not have sufficient resources to practice irrigation farming. As a result of drought, farmers will be forced to adopt drought-resistant crops, which come at higher prices compared to the local landraces where subsistence farmers normally produce their maize [26]. PVABM is thought to be a drought resistant crop [7]. Moussa and Abdel-Aziz [27] found that drought-resistant breeds have different responses to drought. These findings suggest that the response of PVABM to drought is dependent on the drought status of the crop and the environment the plant is grown.

### 3.4. Constraints to Maize Production

#### 3.4.1. Pests

Maize loss to pests remains a huge challenge, especially for low income farmers who have less access to crop protection resources [28]. Losses have an impact on income, thus affecting the food security status of farmers’ households. Sub-Saharan countries have implemented different strategies to combat maize production losses from pests [17]. However, these pests still remain a major challenge, regardless of subsidizing interventions. Stem bores rank among the most troublesome pests for maize producers [28]. They are estimated to cause about 25–45% of the losses during maize cultivation and 30–90% during postharvest and storage. Due to the high losses caused by stem bores, researchers have been prompted to produce resistant maize breeds. However, these resistant hybrids cannot solve the problem, since stem borer can become resistant to them [29]. A good example is that of *Busseola fusca* (Lepidoptera: *Noctuidae*) resistance to *Bacillus thuringiensis* (Bt) maize [30]. The Bt gene does not control the adult larvae, therefore, it has a reduced ability to control stem bores [31]. It can also be argued that smallholder farmers with poor resources struggle to purchase these seeds due to high market prices, and the seeds cannot be recycled due to the reduced yield over a number of years.

#### 3.4.2. Weeds

Striga is one of the most common, problematic weeds in maize production in Africa and it also affects many cereal grain crops [32]. Smallholder farming systems are most vulnerable to the infestation of crops by striga weeds due to the lack of knowledge of how to effectively control this weed and the correct resources to reduce the infestation. Various weeds affect maize production in South Africa and they can be reduced through different control measures.

## 4. Vitamin A Deficiency: A Food and Nutrition Insecurity Challenge 

Child malnutrition, mortality rates and disability are continuous challenges beyond the Millennium Developmental Goals that require urgent attention. Despite various supplementation programmes, pregnant women and children under 5 years remain vulnerable to hidden hunger and vitamin deficiencies, such as VAD [19]. VAD has become more than just a public health problem, as its severe effects have negative implications for child well-being and reduce children’s future potential to actively contribute to the economy of their country. The risks associated with VAD and its effects introduce unjustified expenditure, comparable to if children with VAD were disabled. This results in increased government subsidies, and in South Africa, increases the economic burden of social grants.

**Figure 1:**
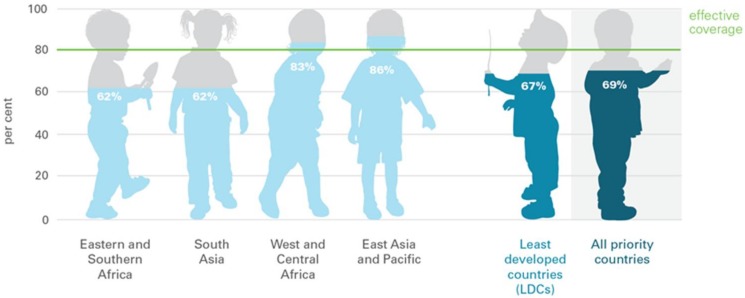
Vitamin A deficiency among the least developed countries (United Nations Children’s Fund (UNICEF)).

United Nations Children's Fund (UNICEF) [33] reported the successful delivery of vitamin A supplements, through the use of integrated child health events, such as child health days, as well as immunization, in some of the least developed countries. Nevertheless, VAD still continues to be a challenge. As shown in Figure 1, there is only 62% of total VAD coverage in eastern and southern Africa.

## 5. Vitamin A Deficiency in South Africa

Pillay et al. [19] confirmed that VAD is an underestimated challenge for the African continent, affecting about 33 million pre-school age children. Malnutrition has been reported to exist in South Africa [34]. Most notable is the rise in micronutrient deficiencies, which are reported as hidden hunger. VAD is a rising micronutrient deficiency that is known to affect children, predominantly. VAD is mostly present in rural communities, where staple crops, such as maize, are consumed on a daily basis. Govender [5] reported that child VAD doubled in South Africa between 1994 and 2006.

Unfortunately, pregnant women and children are the most vulnerable to VAD. In South Africa, the number of people affected by VAD is increasing and high incidences of the deficiency have been reported in rural compared to urban communities [35]. VAD increases the chance of disability and mortality. Despite the achievement of the Millennium Developmental Goals, the VAD situation still remains a significant challenge [36]. In South Africa, there has been no or very little significant difference observed in reducing VAD. The South African government has implemented different strategies to combat VAD in the past 20 years. However, these interventions have had mixed efficacy, which has led to their non-significant impact reducing VAD.

## 6. Remediation Strategies Applied to Reduce Vitamin A Deficiency in South Africa

The South African Government has implemented various strategies to combat VAD, including dietary diversification, vitamin A supplementation and fortification. These strategies have not been successful enough to address VAD for various reasons [37]. Pillay et al. [37] and Govender et al. [22] remark that in South Africa, supplementation programmes and the purchasing of vitamin supplements, which are expensive for low income communities [5], have not been successful, and they recommended an agricultural-based intervention targeting poor communities. The authors further argue that low income communities have limited access to commercial vegetables and animal products that are rich in vitamin A [38]. Consequently, monotonous maize-dominated diets are usually the only meals available and accessible to most poor households.

The national gazette Act No 54 of 1972: Foodstuffs, Cosmetics and Disinfectants mandated a food fortification programme aimed at improving staple crop vitamins and nutrients [39]. This did not work for the country due to the crops being expensive for low income households. Even with this Act, the maize consumed in South Africa had imbalanced nutrients. In South Africa, white maize is predominantly consumed, while yellow maize is used for animal feed. Therefore, the Act was not successful because of consumer perceptions about the product and the crops they consume. Food fortification as an intervention was also susceptible to a number of other factors that hindered its success.

Biofortification is a strategy of addressing VAD and other micronutrient deficiencies in Africa that was introduced by HarvestPlus. The introduction of biofortification as a strategy for addressing VAD in the African continent led to the idea of improving the vitamin A content of maize and sweet potatoes [32]. Biofortified sweet potatoes have already been successfully introduced in South Africa [40], but PVABM has not been introduced as an intervention to VAD. However, the successful introduction of PVABM in other southern African countries, such as Zambia, instils hope for South Africa too.

## 7. Biofortification of New Cultivars for Improved Vitamin A Content

Biofortification is a relatively new agricultural-based strategy to reduce hidden hunger in the SSA region, especially for low income communities [41,42]. Rural communities are the main targeted beneficiaries of PVABM [43]. Biofortification of staple crops ensures improved nutrient supply to poor households though the most preferred diet [7,5,44]. The mineral improvement in biofortified staple crops allows these crops to be resistant to certain plant diseases [45], especially fungal root diseases. Moreover, the planted crops are more likely to survive the seedling stage and their initial growth is faster. Bouis et al. [8] suggested that the biofortification of provitamin A maize improves crops’ ability to resist drought, thus improving the vitamin A composition of the grains. Tumuhimbise et al. [46] also recommended that breeding for biofortification improves drought resistance, vitamin A content and disease tolerance (Table 1). Therefore, PVABM is of great benefit to farmers in rural areas where diseases are a major challenge to plant production and during times where farmers are late planting. PVABM should be easily incorporated because of this potential. In South Africa, sweet potatoes as a biofortified crop have been successfully introduced and well-accepted by consumers of different living standards [39]. However, the level of acceptance for PVABM may differ.

## 8. Provitamin A-Biofortified Maize to Reduce Hidden Hunger: Food for the Future

One of the advantages of PVABM is that it is cheaper compared to other vitamin A supplementations [4]. After crops have been bred and grown, there is a lower cost of production in subsequent years, given the appropriate storage conditions. Moreover, once maize has been produced at the farm level, there is no need for additional fortification or vitamin amendments in people’s diets [37].

Staple crops, such as maize, are used to prepare different meals in rural communities, therefore the improvement of nutrients will stabilize the nutrient composition within them [40]. Biofortification targets staple crops under smallholder farming systems [38]. Different maize products can be produced through PVABM to improve the acceptability and accessibility of vitamin A at the household level. The production of PVABM in rural communities, where maize is used for different products, can improve the local economy through people selling snacks, and can improve food security by allowing different meals to be consumed at different times, resulting in reduced VAD in children. There is no doubt that PVABM would improve the food security status of rural households and alleviate VAD [37]; however, before it can be incorporated into smallholder farming systems, the challenge is the willingness of smallholder farmers to accept PVABM and the acceptability of these products by consumers. Meenakshi et al. [38] argued that the success of PVABM depends on the target population, which are rural or low income people and vulnerable groups (i.e., women and children), and if they accept these varieties or not. These authors also pointed out that rural communities usually confuse yellow maize with orange maize, which could be a major challenge given the perceptions around yellow maize.

PVABM has drawn interest from researchers in different fields across the African continent [12,32], including South Africa. PVABM has the potential to alleviate VAD, hidden hunger and improve food security in rural communities, where the target groups are mostly located. The carotenoid content in PVABM is crucial to addressing VAD.

## 9. Carotenoids in Provitamin A Maize

Maize grain contains different types of carotenoids in the form of provitamin A [22] and are found in yellow and orange maize. Xanthophyll and carotenes result in the carotenoid pigments found in yellow and orange maize and are responsible for the endosperm color (yellow or orange). In PVABM, β-carotene and β-cryptoxanthin have been identified as the most abundant carotenoids, whilst α-carotene is present in smaller capacities [19].

The carotenoid level increases with the color change [18]. Dark orange maize has higher levels of carotenoids compared to other color maize (Table 2), however the orange and dark orange maize are not available for farmers and consumers yet. Pillay et al. [37] argued that color does not really determine provitamin A content in maize because of variable accumulation in the maize kernel (seed coat, endosperm and germ).

Generally, yellow maize contains 0.25 to 2.5 μg/g dry weight (DW) of provitamin A, while PVABM contains higher levels, 15 μg/g DW, of provitamin A [19], but is not yet available on the market.

## 10. Perceptions and Other Factors Influencing the Adoption of Maize Hybrids

Smallholder farmers usual do not adopt improved maize hybrids [47]. The main cause for this could be the lack of consideration of farmers’ preference in the development of these hybrids. Farmers have different preferences and select maize for different traits and the most preferred trait for selection is yield. Farmers should be considered in the production of new hybrids, as their willingness to adopt and incorporate the product is important [39]. Stevens and Winter–Nelson [48] assessed the acceptance of PVABM through taste and trading. Their findings showed that PVABM was accepted by consumers regardless of its orange color. However, these findings may vary between countries and regions. Nuss et al. [42] observed similar findings. In their study, they observed that in Zambia, children easily adapt to orange maize (PVABM). However, this may not be the case amongst older groups [8] due to social pressures and diet. Pillay [19] found that PVABM had the potential to alleviate VAD in Kwazulu-Natal, and their findings showed that orange PVABM maize was accepted by consumers, although white maize remained the most preferred maize type by adults and high school children. Govender et al. [22] found that PVABM porridge was deemed to be acceptable by caregivers in Kwazulu-Natal. These findings show that preparing diverse meals and products can improve the acceptability of PVABM by consumers, thus reducing hidden hunger at different age levels. Beswa et al. [49] suggested that PVABM mixed with amaranth leaf powder has the potential to produce a nutrient rich snack; however, the acceptability of these snacks by consumers is of concern.

De Groote et al. [17] suggested that maize preference plays a major role in maize selection and that these attitudes are regional. Moreover, yellow maize in South Africa is believed to have less of an acceptable taste and is considered a drought crop [50,38]. Yellow maize is perceived for animal consumption rather than human consumption, while white maize is used primarily for human consumption [38]. The orange color of PVABM could lead to farmers recommending it for animal consumption [17,51]. These perceptions can be changed through the provision of breeding information and education regarding the benefits of orange maize. Alternatively, Odunitan-Wayas et al. [35] reported that PVABM can be fed to indigenous and layer chickens as a way of improving vitamin A consumption through meat and egg consumption. Furthermore, their study showed that sensory characteristics had no influence on consumer preference. Animals can be used as a secondary source of vitamin A after feeding them biofortified maize, to balance diets [36]. The authors further suggest that this strategy could improve the production of PVABM for both human and animal consumption.

In southern African countries, the price of yellow maize is less than the price of white maize due to consumer preferences and the yellow maize market [22]. Therefore, questions remain as to whether perceptions and prices of yellow maize could have an impact on PVABM. The low price of orange maize could encourage farmers to adopt the variety and incorporate it into their smallholder farming systems [37]. Moreover, this could have economic benefits for smallholder farmers, given the availability of the PVABM market for selling excess maize, should they have a surplus. In Zimbabwe, smallholder farmers have been found to be more willing to pay for maize varieties that improve the food security in their household and generate income [25]. One major reason leading to smallholder farmers not adopting new hybrids, such as PVABM, is the lack of trust farmers have of new breeds. Previous studies in the southern African region have shown that consumers and smallholder farmers are willing to adopt biofortified products, including PVABM [50,11,37]. This could be an advantage for the production of biofortified crops, especially in areas where communities have doubts about genetically modified (GMO) crops. More so, willingness to adopt PVABM could be associated with education regarding the impact of maize cross pollination on the local landraces used by smallholder farmers in rural communities [38], since in South Africa, most subsistence farmers still use local maize landraces [15].

## 11. Provitamin A-Biofortified Maize as Income for Smallholder Farmers

The acceptability of PVABM by consumers of all standards will allow a market for farmers, especially subsistence rural farmers in areas where VAD is prevalent. The willingness to pay for these new maize varieties would create income for low income houses [25], while also diversifying peoples’ diets. However, several factors may impact the successful marketing of PVABM, such as sensitivity to color and taste, and the agronomic potential of these varieties. Zuma et al. [52] noted that farmers in the Kwazulu-Natal province of South Africa perceived PVABM as similar to common yellow maize. The authors further noted that smallholder farmers would accept PVABM for consumption but mostly they would want to produce it to sell since these varieties are still new in this country. These findings suggest that PVABM can be incorporated into a rural market, for both animal and human consumption.

## 12. Conclusions

PVABM has the potential to reduce VAD in rural South African communities and to aid in alleviating the hidden hunger experienced by low income households. As a biofortified crop, it has improved resistance to drought and disease, therefore its production would improve yields in areas where drought is a challenge. PVABM acceptance relies on its successful introduction to farming systems in rural communities and its potential yield under different climatic conditions. Although PVABM has the potential to alleviate hidden hunger in rural communities, the challenge is in the introduction of these varieties to smallholder farming systems, where local landraces are the dominant maize varieties grown. Additionally, farmers will have to trust the PVABM variety before incorporating it into their crop farming system because of its color. Previous studies have shown that farmers are sensitive to yellow maize and their belief is that yellow maize is for animal feeding. Should there be a willingness to incorporate PVABM crops then farmers will need to be convinced they have higher yields compared to white maize crops and the local landraces they usually produce. Information workshops on the importance of vitamin A and the challenges of VAD to smallholder farmers and their consumers could aid in the acceptance of PVABM in low income communities. This would help to build capacity around the sustainable production of PVABM for the alleviation of VAD and the improvement in nutrient intake by rural households. Collaborative research involving different stakeholders, such as researchers, government agencies, farmers and NGOs, is needed to research current farming systems and the importance of those systems.

## Figures and Tables

**Table 1:** Different biofortified staple crops in different countries with their agronomic and micronutrient performance.

Staple Crop	Targeted Nutrient	Targeted Country	Agronomic Traits
Bean	Iron	DR Congo, Rwanda	Virus resistance, heat and drought tolerance
Cassava	Vitamin A	DR Congo, Nigeria	Disease resistance
Maize	Vitamin A	Nigeria, Zambia	Disease resistance, drought tolerance
Pearl Millet	Iron	India	Mildew resistance, drought tolerance
Rice	Zinc	Bangladesh, India	Disease and pest resistance, cold and submergence tolerance
Sweet Potato	Vitamin A	Mozambique, Uganda, South Africa	Disease resistance, drought tolerance, acid soil tolerance
Wheat	Zinc	India, Pakistan	Disease and lodging resistance

Source: [6,16].

**Table 2:** Carotenoid concentrations for different maize varieties (white, yellow, orange and dark orange).

	Carotenoids (nmol/g)				
Maize	Lutein	Zeaxanthin	*β*-cryptoxanthin	*β*-carotene	*β*-carotene
White	1.1 ± 0.01	0.09 ± 0.01	-	-	0.05 ± 0.002
Yellow	16.8 ± 0.6	5.4 ± 0.5	2.6 ± 0.4	0.44 ± 0.04	0.77 ± 0.14
Orange	15.7 ± 0.3	11.6 ± 0.3	5.4 ± 0.05	0.58 ± 0.02	5.6 ± 0.1
Dark orange	19.1 ± 4.5	11.8 ± 2.9	5.3 ± 0.7	1.53 ± 0.04	13.9 ± 0.7

Source: [18].
